# Comparative efficacy of anthelmintics and their effects on hemato-biochemical changes in fasciolosis of goats of South Gujarat

**DOI:** 10.14202/vetworld.2016.524-529

**Published:** 2016-05-28

**Authors:** R. G. Shrimali, M. D. Patel, R. M. Patel

**Affiliations:** Department of Veterinary Medicine, College of Veterinary Science & Animal Husbandry, Navsari Agricultural University, Navsari - 396 450, Gujarat, India

**Keywords:** anthelmintics, efficacy, fasciolosis, goats, hemato-biochemical changes

## Abstract

**Aim:**

Fasciolosis is a parasitic disease caused by *Fasciola* spp. of the family *Fasciolidae (trematodes)* characterized by bottle jaw, anemia, progressive debility, and potbelly condition. There are many aspects of fasciolosis remaining unknown thus hemato-biochemical alterations in closantel, triclabendazole + ivermectin, and oxyclozanide + levamisole treated goats were studied.

**Materials and Methods:**

A total of 40 naturally fasciolosis infected goats having egg per gram more than 100 were randomly divided into four groups. Goats of Group I-III were treated with three different anthelmintics, whereas, goats of Group-IV were kept as control or untreated. Whole blood, serum, and fecal samples were collected on 0, 7^th^, and 30^th^ day of treatment.

**Results:**

During the study, values of hemoglobin, total erythrocyte count, pack cell volume, and total protein were significantly elevated to their normal levels in anthelmintics treated groups. Whereas, values of total leukocyte count, aspartate transaminase (AST), lactate dehydrogenase (LDH), and gamma-glutamyl transferase (GGT) were significantly reduced to their normal level in anthelmintics treated groups. The efficacy of closantel (T1), triclabendazole + ivermectin (T2), and oxyclozanide + levamisole (T3) was 99.63%, 100%, and 94.74% and 100%, 100%, and 97.38% on 7^th^ and 30^th^ day of treatment, respectively.

**Conclusions:**

Fasciolosis in goats can be diagnosed on the basis of fecal sample examination, but alterations in important biomarkers such as AST, GGT, and LDH are also helpful for early diagnosis. The use of newer anthelmintic either alone or in combination showed a higher therapeutic response in fasciolosis of goats.

## Introduction

Parasitic infections pose a serious health threat and limitation to the productivity of small ruminants due to the associated morbidity, mortality, cost of treatment, and control measures. Among various parasitic infections of small ruminants, fasciolosis is the most important pathogenic parasitic infection which is widely distributed in India causing severe infection, anemia, and hypoalbuminemia. The prevalence of fasciolosis largely depends on rainfall and production systems [1].

The pathogenic effects of these flukes on the host organism begin with the ingestion of encysted metacercaria with vegetation or freshwater. After migration of juvenile forms through the hepatic parenchyma, flukes reside and graze on the mucosa of the bile ducts, which result in the massive tissue damage. The lesions in the liver are only partially a result of the mechanical action of liver fluke because the injury of the liver can be induced by parasites excretory products, decomposed products of parasites, bile, and hepatic tissue. Pathological changes, caused by mechanical and toxic effects of *Fasciola hepatica*, affect the complex vascular and biliary system in the liver. Properly functioning of these two systems is the most important factor for preservation of normal liver functions. In complexity of patho-physiological examination, the determination of serum transaminases, (alanine aminotransaminase [ALT] and aspartate transaminase [AST]) which are the most sensitive indicator of hepatocellular injury. Further, elevation in alkaline phosphatase (ALP), lactate dehydrogenase (LDH), gamma-glutamyl transferase (GGT), serum proteins, and bilirubin having significant importance in the degree of cholestasis and synthetic capacity of the liver [2,3].

A wide range of broad-spectrum anthelmintics are available for use in cattle, sheep, and goats including the benzimidazoles, the avermectins, tetrahydropyrimidines, and imidothiazoles, which are having varying efficacy against parasitic gastroenteritis and bronchitis. The activity of each of these drugs is variable against the different stages of the parasites’ development. Some, like triclabendazole are very effective against immature flukes, whereas others, *viz*., nitroxynil or closantel, are highly efficient against adults [4]. Fasciolosis may be controlled with the salicylanilide and related phenolic compounds. Salicylanilides are effective against a wide range of hepatic and intestinal trematodes in a variety of animals [5]. The later being the first fasciolicide with excellent activity against all stages of *Fasciola hepatica* infection. The implication of strategies for Helminth’s resistance and individual immunity require periodic evaluation of these drugs. During this study, three anthelmintics claimed to be effective against fasciolosis were evaluated by studying their effects on important hemato-biochemical parameters in fasciolosis infected goats in the absence of such systemic studies on fasciolosis in goats in study areas.

## Materials and Methods

### Ethical approval

The prior approval from the Institutional Animal Ethics Committee was obtained for use of farmer’s animals in this study.

### Selection of animals

**Figure-1:**
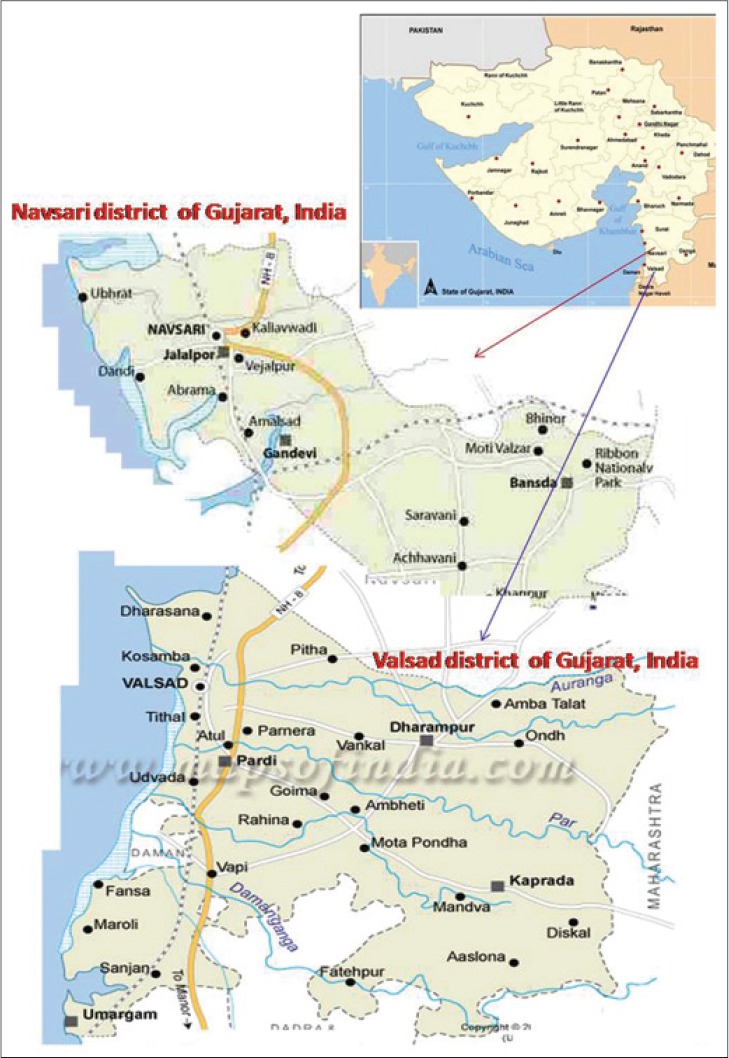
Area covered under the research work.

During an epidemiological survey, on gastrointestinal parasites in goats of Navsari and Valsad districts of south Gujarat (Figure-1), goats with poor body condition, history of diarrhea, anemia were suspected to be having parasitic infection and were screened for the presence of parasitic ova. Goats those having *Fasciola* spp. infection and egg per gram (EPG) for more than 100/g of fecal sample were included for evaluating anti-fasciolosis drugs. A total of 40 naturally fasciolosis infected local goats between 1.5 and 3 years of age and 20-35 kg body weight were included. They were randomly divided into four treatment groups, i.e. Group-I: Closantel, Group-II: Triclabendazole + ivermectin, Group-III: Oxyclozanide + levamisole, and Group-IV: Control or untreated. The detailed treatment protocol is given in Table-1.

**Table-1:** Treatment protocols to study the efficacy of various anthelmintics in fasciolosis infected goats of South Gujarat.

Groups	Treatment protocols	Dose rate	Number of animals	Mean EPG
T1	Closantel	15 mg/kg body wt. PO	10	110.90
T2	Triclabendazole+ivermectin	10.2 mg/kg body wt. PO	10	110.00
T3	Oxyclozanide+levamisole+silymarin	10 mg/kg body wt. PO	10	110.80
T4	Control group	No treatment	10	110.80

EPG=Egg per gram

### Sample collection and processing

Fecal, whole blood, and serum samples from goats of all treatment groups were collected at 0, 7^th^, and 30^th^ day of treatment. Fresh fecal samples were collected directly from the rectum of goats and kept immediately in plastic containers containing 10% formalin for preservation until used for examination. The qualitative examination was carried out for the presence of parasitic eggs/oocysts under ×10 magnifications under a microscope for best identification. Quantitative examinations of fecal samples of *Fasciola* spp. were carried out within 48 h of collection using a modified McMaster’s technique to count EPG [6].

Blood samples for hematological and biochemical analysis were collected separately into 6 ml capacity marked vacutainers and transported to the laboratory at 4°C in pre-cooled ice-box. Serum was separated out and stored at −20°C till the further use. Hemoglobin (Hb, g%), packed cell volume (PCV, %), total erythrocyte count (TEC, × 10^6^/cu.mm), and total leukocyte count (TLC, × 10^3^/cu.mm) were estimated in fully automatic hematology cell counter (Exigo Vet, Sweden) on the same day. Whereas, various biochemical parameters, *viz*., total protein (TP, g/dl), aspartate transaminase (AST, IU/L), ALT (IU/L), LDH (IU/L), and GGT (IU/L) were estimated in semi-automatic biochemistry analyzer (Microlab 300, Merck, Netherland) using commercially available diagnostic kits (Coral Clinical System, Goa) within a day or two.

### Statistical analysis

Data were statistically analyzed using IBM SPSS statistical software version 20.0. Significant differences between days within treatment and between treatment at different times were determined by one-way ANOVA test for variance analysis at p<0.05 [7]. The descriptive data are presented as the means ± standard error. The efficacy of anthelmintics was evaluated based a formula [8]:

% of drug efficacy = P-R/P×100

Where, R = Average number of parasite egg in a gram of fecal sample after treatment,

P = Average number of parasite egg in a gram of fecal sample before treatment.

## Results and Discussion

### Hematological alterations in different treatment groups

The changes in different hematological parameters at different time intervals in various treatment groups are given in Table-2. The mean Hb concentrations in treatment groups (T1, T2, T3, and T4) was comparatively lower than normal values in goats (8-12 g%). Similarly, lower Hb concentration in *Fasciola* spp. infected animals was also reported (9-12). At day 7, the mean Hb concentrations were increased significantly in anthelmintics treated groups (T1, T2, and T3) as compared to control group (T4) (p<0.01). The mean Hb values were further significantly increased in T1, T2, and T3 (p<0.01) on the 30^th^ day of anthelmintic treatment. The findings of increased Hb concentrations in anthelmintic treated goats are in accordance with previous report [9] of increased Hb concentrations to normal level in anthelmintic treated animals in the treatment group at 28^th^ day post-treatment.

**Table-2:** Hematological changes in various treatment groups at different time intervals in fasciolosis of goats.

Parameters	Time intervals	Treatment groups				F value

		T1	T2	T3	T4	
EPG	0	110.90±0.32^y^	110.00±0.36^y^	110.80±0.21^z^	110.80±0.32^x^	0.10
	7	0.40±0.30^×a^	0.00±0.00^×a^	5.80±1.36^ya^	236.90±6.01^yb^	1445.67**
	30	0.00±0.00^×a^	0.00±0.00^×a^	2.90±0.92^×a^	347.80±10.14^zb^	1158.90**
F value		61162.40**	90750.00**	4044.83**	302.90**	
Hb (g %)	0	6.70±0.05^×c^	6.56±0.09^×c^	4.93±0.05^×a^	5.79±0.18^a^	55.68**
	7	8.01±0.04^yd^	7.73±0.03^yc^	6.82±0.04^yb^	5.63±0.17^a^	123.07**
	30	9.00±0.03^zd^	8.72±0.03^zc^	8.82±0.05^zbc^	5.51±0.14^a^	387.09**
F value		679.36**	267.28**	1393.85**	0.68	
PCV (%)	0	18.90±0.27^×c^	18.32±0.40^×c^	14.70±0.15^×a^	16.11±0.27^b^	44.51**
	7	24.02±0.15^yb^	18.96±2.75^×a^	19.85±0.22^ya^	16.27±0.44^a^	5.24*
	30	27.10±0.11^zb^	26.69±0.35^yb^	26.86±0.28^zb^	16.05±0.33^a^	354.32*
F value		450.79**	8.24**	729.47**	0.102	
TEC (×10^6^/cu.mm)	0	5.72±0.05^×b^	5.43±0.07^×a^	5.38±0.04^×a^	5.29±0.01^ya^	12.42**
	7	5.96±0.03^yc^	5.94±0.03^yc^	5.78±0.03^yb^	5.22±0.03^ya^	98.04**
	30	8.56±0.10^zb^	8.70±0.05^zb^	8.72±0.04^zb^	5.07±0.04^×a^	675.04**
F value		463.81**	903.43**	1919.15**	10.46**	
TLC (×10^3^/cu.mm)	0	12.55±0.17^za^	12.67±0.13^za^	13.16±0.07^zb^	12.61±0.17^a^	3.64*
	7	8.47±0.17^ya^	8.98±0.03^yb^	9.24±0.14^yb^	12.79±0.15^c^	205.13**
	30	4.80±0.04^×a^	4.67±0.05^×a^	4.91±0.03^×a^	12.94±0.17^b^	1848.05**
F value		745.13**	2083.08**	1902.36**	0.94	

Means with different superscripts a, b, c, and d along a row differ significantly at p<0.01, Means with different superscripts x, y, and z along a column differ significantly at p<0.01,

** Highly significant at p<0.01,

* Significant at p<0.05. EPG=Egg per gram, Hb=Hemoglobin, PCV=Packed cell volume, TEC=Total erythrocyte count, TLC=Total leukocyte count

Initially, the mean PCV values in all treatment groups (T1, T2, T3, and T4) were lower than normal range (22-38%). The present findings are in agreement with previous reports of lower PCV values in *Fasciola* spp. infected animals [10-13]. On 7^th^ day, the mean PCV increased significantly in T1 and T3, whereas the increase in mean PCV value was non-significantly in the T2 group. Further, a significant increase in PCV was also observed in T1, T2, and T3 from day 7 to day 30 (p<0.01). A similar trend of increase in PCV in anthelmintic treated animals at 7^th^ and 28^th^ day post-treatment was also observed by Khalil *et al*. [9]. In the control group (T4), the mean PCV remained lower than normal range of PCV at different time intervals. The lower PCV could be attributed to an abnormal loss of red blood cells due to feeding habits of flukes or to an excessive destruction of RBCs caused by some hemolyzing factors produced by the flukes Okoye *et al*. [13].

The mean TEC was comparatively lower in all treatments groups as compared to normal TEC level (8-18 × 10^6^/cu.mm) in goats. The results are in accordance with the findings of previous reports, in which lower TEC in fasciolosis affected animals was observed [10-13]. On the 7^th^ day of treatment, the mean TEC increased significantly in T1, T2, and T3 than the control group (T4) (p<0.01). Subsequently, increasing trend in TEC in anthelmintic treated groups (T1, T2, and T3) was also observed on 30^th^ day of treatment. A similar trend of increasing TEC in goats treated for fasciolosis was observed by Khalil *et al*. [9], whereas in control group decreasing trend in TEC was observed from day 0 to day 30. The decrease in TEC in fasciolosis affected animal could be attributed to chronic blood loss due to the blood-sucking activity of the adult flukes and leakage of blood from the bile duct to the intestine Okoye *et al*. [13].

During the study, the mean TLC was higher in all treatment groups on day 0 as compared to normal range (4-11 × 10^3^/cu.mm) in goats. A similar trend of higher TLC in fasciolosis infected animals was also observed in previous reports [10,11,13]. The mean TLC was decreased significantly in T1, T2, and T3 from day 0 to day 30 (p<0.01), whereas the mean TLC values remained static without significant difference over time intervals in the control group (T4).

### Biochemical alterations in different treatment groups

*F. hepatica* causes the release of reactive oxygen which result in damage of cell wall and hepatic tissue necrosis [11]. These changes have an influence on biochemical parameters in serum, and determination of specific liver enzymes is very valuable tool for diagnosis of hepatobiliary diseases. Physiologically, the normal levels of the enzymes in cells or serum are maintained by constant synthesis, simultaneous degradation, inactivation, and elimination of enzymes [9-11]. However, due to disruption of hepatocellular integrity, enzymes from damaged cells are released into the blood serum and their concentration increase/decrease above the physiological values. The changes in certain biochemical parameters at different time intervals in fasciolosis infected goats under different treatment groups are given in Table-3.

**Table-3:** Biochemical changes in various treatment groups at different time intervals in fasciolosis of goats.

Parameters	Time intervals	Treatments				F value

		T1	T2	T3	T4	
AST (IU/L)	0	313.11±4.70^za^	304.9±5.19^za^	331.75±3.23^zb^	309.50±7.04^a^	5.68*
	7	295.51±1.92^yab^	280.90±2.78^ya^	280.60±11.12^ya^	308.84±6.24^b^	4.193*
	30	140.90±1.90^×a^	146.83±2.15^×a^	147.69±2.09^×a^	315.96±4.61^b^	861.86**
F value		915.09**	552.62**	195.42**	2.10	
ALT (IU/L)	0	44.99±1.31	42.71±0.34	43.31±0.23	43.62±0.70	3.85*
	7	45.13±0.69	43.01±0.36	43.66±0.22	44.69±1.34	1.50
	30	45.83±0.86	38.97±3.79	43.17±0.46	44.92±1.21	2.195
F value		0.205	1.035	0.59	1.92	
TP (g/dl)	0	4.03±0.04^×c^	3.79±0.03^×b^	3.50±0.10^×a^	3.61±0.06^ab^	11.10**
	7	4.64±0.10^yc^	4.21±0.02^yb^	4.28±0.03^yb^	3.65±0.09^a^	32.52**
	30	6.39±0.0^zc^	6.13±0.06^zb^	6.08±0.12^zb^	3.54±0.09^a^	225.77**
F value		282.56**	809.68**	189.69**	0.42	
LDH (IU/L)	0	2287.88±14.31^zc^	2049.67±26.33^ya^	2301.06±4.50^zc^	2146.56±39.58^b^	23.24**
	7	2009.24±1.55^yab^	1921.08±79.31^ya^	2068.15±12.85^ybc^	2154.63±40.05^c^	4.7*
	30	1186.29±15.59^×ab^	1138.53±14.40^×a^	1213.50±17.69^×b^	2161.47±40.60^c^	401.31**
F value		2183.98**	101.42**	1972.49**	0.035	
GGT (IU/L)	0	231.08±1.91^zb^	219.29±1.43^za^	227.60±1.83^zb^	227.26±1.95^×b^	7.70**
	7	105.32±1.22^ya^	107.19±1.27^ya^	113.06±1.21^yb^	231.30±2.13^×yc^	1652.22**
	30	53.71±0.99^×a^	51.26±0.868^×a^	49.63±1.70^×a^	234.52±1.86^zb^	4115.53**
F value		4068.77**	4946.15**	3157.55**	3.34*	

Means with different superscripts a, b, c, and d along a row differ significantly at p<0.01, Means with different superscripts x, y, and z along a column differ significantly at p<0.01,

* Highly significant at p<0.01,

** Significant at p<0.05. AST=Aspartate transaminase, LDH=Lactate dehydrogenase, GGT=Gamma-glutamyltransferase, ALT=Alanine aminotransaminase, TP=Total protein

During the study, initial mean AST levels in all treatment groups were comparatively higher (304.9-331.75 IU/L) as compared to normal level (66-230 IU/L) in goats. The present findings are in accordance with previous reports [9,14-16]. The mean AST level decreased significantly from day 0 to day 7 and further from day 7 to day 30 in T1, T2, and T3 (p<0.01). Similarly, decrease in mean AST in animals treated for fasciolosis was also reported by Khalil *et al*. [9]. The mean AST levels in the untreated group (T4) remained constantly higher or static with the non-significantly difference between time intervals. The enzyme AST is a sensitive indicator of parenchymal damage of the liver. The significant elevation of AST indicates chronic fasciolosis in infected goats. Higher levels of AST suggest lack of hepatocellular damage and probably indicate a chronic fasciolosis (biliary phase) and regenerative changes in the parenchyma [11]. The means ALT levels in all treatment groups was also higher than its normal range (7-24 IU/L). However, the mean ALT levels in different treatment groups (T1, T2, T3, and T4) at different time intervals (day 0-7 and day 7-30) varied from each other but difference was non-significant which indicates chronic fasciolosis [14,15].

In this study, the mean TP levels in all treatment groups were comparatively lower than normal range (6.4-7.8 g/dl). The decrease in total serum protein in fasciolosis infected animals was also reported in previous reports [14-17]. In fasciolosis, decreased TP level is suggestive of loss of plasma protein through bile ducts into intestine as a result of increased permeability of the hyperplastic bile duct epithelium and loss of plasma proteins through the fluke’s digestive tract [11,15]. The mean TP levels showed a significantly increasing trend in T1, T2, and T3 from day 0 to day 30, whereas in the control group (T4), it remained more or less static. Significantly increase in TP levels in fasciolosis infected animals after treatment was also reported by Sheikh *et al*. [15], whereas Khalil *et al*. [9] observed a non-significant increase in TP in anti-fasciolosis anthelmintics treated animals.

The mean LDH levels in treatment groups were highly elevated than normal range (78-265 IU/L) before initiation of treatment. The findings are accordance with previous reports which reported increased LDH level in animals in fasciolosis infection. Increases levels of LDH are related to the inflammatory state of liver and tissue destruction provoked by the parenchymal migration of juvenile flukes [9,11,16]. Thereafter, mean LDH levels in T1 and T3 decreased significantly from day 0 to day 7 (p<0.01), whereas the value decreased from 2049.67 to 1921.08 (IU/L) in T2 at 7^th^ day but the difference was non-significant. Further, mean (LDH) levels in T1, T2, and T3 decreased significantly from 7^th^ to 30^th^ day (p<0.01). The results are in accordance with findings of the previous report of Khalil *et al*. [9], who had reported a significant decrease in the level of LDH in fasciolosis infected animals on the 14^th^ and 28^th^ day post-treatment (p<0.05). In the control group (T4), the LDH level remained at higher side throughout trail period.

The mean GGT levels were comparatively higher as compared to normal range (20-50 IU/L) in all treatment groups of goats before initiation of drug trial. Similarly, higher GGT was observed in fasciolosis infected animals in previous reports [9,10,14-17]. Thereafter, significantly decreasing trend was observed in anthelmintic treated groups (T1, T2, and T3) on day 7 and day 30, whereas the mean GGT level in control animals increased significantly (p<0.05) from day 0 (227.26) to day 30 (234.52). The damage to the bile duct epithelium causes the release of GGT into circulation. This enzyme increases its level in serum mainly after flukes entered in the bile duct. The elevated levels of GGT were an indicator of epithelial damage in the bile duct. The marked increase in GGT level associated with cholestasis and bile duct damage [10,11].

### Efficacy of anthelmintics

Based on EPG, the efficacy of closantel and triclabendazole + ivermectin was about to cent percent at 7^th^ and 30^th^ day of treatment (Table-4). The results are in accordance with the observations of previous reports [18-22], whereas the efficacy of oxyclozanide + levamisole was 94.74% and 97.83% at 7^th^ and 30^th^ day of treatment, respectively. In context to efficacy of oxyclozanide + levamisole, an earlier report of Ratnaparkhi *et al*. [23] reported higher efficacy (100%) in *Fasciola* spp. infections. However, in a recent report of Tadesse *et al*. [22] recorded only 28.14% efficacy of these drugs in *Fasciola* spp. infections of small ruminants. The reduction in efficacy of oxyclozanide + levamisole may be due to anthelmintic resistance as result of prolonged and repeated use of the same drug [24].

**Table-4:** Efficacy of different anthelmintics drugs in fasciolosis of goats.

Particulars	Efficacy (%)	

	On 7^th^ day	On 30^th^ day
T1 (closantel)	99.63	100
T2 (triclabendazole+ivermectin)	100	100
T3 (oxyclozanide+levamisole)	94.74	97.38
T4 (control)	-		

## Conclusions

Activities and concentration of AST, ALT, GGT, and LDH in serum are reliable indicators of fasciolosis and could be used as biomarkers for early diagnosis and to test the effectiveness of anthelmintic therapy. Use of newer anthelmintics showed a higher therapeutic response in fasciolosis of goats. Therefore, it can be concluded that the knowledge of epidemiology and ecology of the parasites is needed not only for planning better strategies of fasciolosis but also provide insight into the natural processes of controlling parasite population. Further, an early diagnosis and treatment with newer drugs for fasciolosis in goats could be advised to reduce economic losses. The results of this study can be useful to future research and planning out proper control of parasitic infections in goats of South Gujarat.

## Authors’ Contributions

RGS carried out the study and tabulated and analyzed the data. MDP analyzed data and drafted the manuscript and revision of the manuscript. RMP rendered necessary infrastructure facilities and revision of the manuscript. All authors read and approved the final manuscript.
